# Barriers, facilitators, and opportunities to promote healthy weight behaviors among preschool-aged children in two rural U.S communities

**DOI:** 10.1186/s12889-022-14770-w

**Published:** 2023-01-07

**Authors:** Katherine Jochim Pope, Cason Whitcomb, Maihan Vu, Lisa Macon Harrison, Joel Gittelsohn, Dianne Ward, Temitope Erinosho

**Affiliations:** 1 Department of Applied Health Science, Indiana University Bloomington, Bloomington, IN 47405 USA; 2 FHI 360, 359 Blackwell Street, Suite 200, Durham, NC 27701 USA; 3 Department of Health Behavior and Center for Health Promotion and Disease Prevention, University of North Carolina at Chapel Hill, Chapel Hill, NC 27599 USA; 4 Granville Vance Public Health Department, 115 Charles Rollins Road, Henderson, NC 27536 USA; 5 Department of International Health, Johns Hopkins University, Baltimore, MD 21205 USA; 6 Department of Applied Health Science, Indiana University Bloomington, 1025 East 7th Street, Bloomington, IN 47405 USA

**Keywords:** Childhood obesity, Nutrition, Physical activity, Rural, Community engagement, Preschool-aged children

## Abstract

**Background:**

Obesity levels are higher in rural versus urban children. Multi-level community-based interventions can be effective in promoting healthy child weight, but few of such interventions have focused on rural children. This formative study assessed barriers, facilitators, and opportunities to promote healthy child weight in two rural communities.

**Methods:**

Multiple data collection methods were used concurrently in two rural communities in Indiana and North Carolina. Focus groups and interviews were conducted with participants, including parents of children aged 2–5 years (*n* = 41), childcare providers (*n* = 13), and stakeholders from 23 community organizations. Observational audits were conducted at 19 food outlets (grocery stores) and 50 publicly-accessible physical activity resources. Focus groups/interviews were analyzed thematically. Surveys were analyzed using descriptive statistics, Fisher’s exact test, and t-tests.

**Results:**

Family level barriers included limited financial resources and competing priorities, whereas parental role-modeling was perceived as a facilitator of healthy weight behaviors. At the organizational level, childcare providers and community stakeholders cited limited funding and poor parental engagement in health promotion programs as barriers. Childcare providers explained that they were required to comply with strict nutrition and physical activity guidelines, but expressed concerns that similar messages were not reinforced at home. Facilitators at the organizational level included healthy meals provided at no cost at childcare programs, and health promotion programs offered through community organizations. At the community level, lack of public transportation, and limited access to healthy food outlets and physical activity-promoting resources posed barriers, whereas existing physical activity resources (e.g., parks) and some ongoing investment to improve physical activity resources in the community were assets. In designing/implementing a potential child obesity prevention intervention, participants discussed the need to garner community trust, emphasize wellness instead of obesity prevention, establish community partnerships, and leverage existing community resources.

**Conclusions:**

Rural areas experience multiple challenges that make it difficult for children/families to engage in healthy weight behaviors. This study highlights several assets (existing programs/resources, expertise within communities) that can be leveraged as facilitators. Findings will guide the study team in developing a child obesity prevention intervention for the two rural communities.

## Introduction

One in five Americans live in rural areas [1]. Positive aspects of rural life include cultural values that iterate the importance of kinship [2, 3], and the proximity to natural landscapes that provide opportunities for outdoor activities (e.g., fishing, camping) [3]. Rural residents demonstrate pride for their home, social cohesion, and cross-sector collaboration [4]. However, rural residents also experience health challenges, including childhood obesity, that disproportionately affects rural versus urban children [5]. The etiology of childhood obesity in rural areas is complex [5], influenced in part by unhealthful dietary intake and low levels of physical activity (PA) [5, 6]. Family socioeconomics influence food and PA opportunities available to children [7], with nearly one-quarter (21%) of rural children living in low-income households [8]. Rural areas tend to be food deserts with fewer full-service food outlets and limited access to PA resources [9, 10]. Lack of obesity prevention policies is also a challenge in many rural communities [11].

Given the myriad factors that contribute to childhood obesity, there has been a growing emphasis on multi-level community-based approaches to prevent childhood obesity, as opposed to interventions that target change at a single level of influence such as the child or family [12]. Multi-level community-based interventions expose entire communities to child obesity prevention efforts and target change at multiple levels of influence simultaneously [13], including the child (e.g., through implementation of nutrition and PA lessons); family (e.g., through parenting classes, and provision of reduced-price coupons to facilitate access to healthy foods); organizational settings (e.g., improvements to school food service, and provision of play equipment to support PA); and the broader community (e.g., through social marketing campaigns, and partnerships with community organizations to implement nutrition and PA programs/events) [14–17]. Multi-level community-based interventions have been shown to be effective in promoting sustainable, long-term improvements in children’s weight [15–18]. To date, few multi-level childhood obesity prevention studies have been conducted in the U.S. (e.g., Shape Up Somerville, Healthy Living Cambridge Kids, B’More Healthy Communities), but none have focused on rural preschool children aged 2–5 years old [15–18].

This paper describes barriers, facilitators, and opportunities to promote healthy dietary and PA behaviors in preschool children aged 2–5-years in two rural, underserved U.S. communities with high obesity levels. This formative research was conducted to guide the development of a community-based intervention to prevent childhood obesity.

## Methods

### Study setting and participants

This formative study was implemented between June 2019 and August 2021 in a rural community in Indiana (“IN community” hereon) and North Carolina (“NC community” hereon), respectively. Criteria for selecting communities included: having high child poverty and obesity levels; and the convenience of data collection. Both communities have a high prevalence of child poverty, ranging between 16%-36% [19, 20]. In the NC community, about 20% of preschool-aged children (i.e., 1 in 5) are obese [21]. While there are no obesity estimates for children in the IN community, approximately 36% of adults in the IN community are obese [20]. Given existing research that shows that children often mimic their parents’ obesity status [22], we hypothesized that obesity prevalence for preschool-aged children in the IN community would be similar to the high obesity levels in adults. Ease of data collection was also considered, given that the study team had existing collaborations with stakeholder organizations in the two communities. Notably, the communities differ in racial/ethnic make-up, with IN being predominantly White (98%) [23], while the NC community is diverse (52% Black; 8% Hispanic/Latino) [24].

Convenience sampling was used to recruit parents of children aged 2–5 years, childcare providers, and community stakeholders across both communities. Eligible parents had to be the primary caregiver for a child aged 2–5 years and must have resided in the target communities for ≥ 6 months. Potential parent participants were recruited through flyers posted at community locations, social media, and word-of-mouth from partners at community organizations. Childcare providers (“providers” hereon) included owners/directors, and teachers at childcare centers and family childcare homes. Potential providers were identified from databases obtained from the respective states’ early care and education agencies [25, 26] and recruited through telephone calls. Community stakeholders (“stakeholders” hereon) comprised of policymakers (e.g., county manager, health department director) and representatives of community organizations that served children/families (e.g., Special Supplemental Nutrition Program for Women, Infants and Children (WIC), housing authorities, faith-based organizations). Potential stakeholders were identified through their organizations’ websites and recruited using telephone calls/emails, with snowball sampling used to identify additional stakeholders.

Across both communities, participants included 41 parents (14 in IN, and 27 in NC), 13 childcare providers (7 in IN, and 6 in NC), and stakeholders from 23 community organizations (12 in IN, and 11 in NC). Participants received a $25-$30 gift card to a local grocery store to thank them for their participation. The study procedures were approved by the Institutional Review Boards at the University of North Carolina and Indiana University Bloomington. Informed consent was obtained from participants before data collection.

### Data collection

Guided by the Socioecological Model [27], the study team assessed barriers, facilitators, and opportunities to promote healthy weight behaviors at the interpersonal (family), organizational (childcare programs, community organizations), community (built/local food and PA environment) and policy levels. Multiple methods were used concurrently to collect data, including focus group discussions with parents; interviews with parents, providers, and stakeholders; and observational audits of the built food and PA environment.

Parents in the NC community participated in focus group discussions (4 focus groups; 27 parents total) that occurred in-person at community locations before the COVID-19 pandemic occurred (between September 2019 and March 2020), whereas in IN, one-on-one interviews were held with parents (14 parents total) by telephone or video call (Zoom) because data collection in IN occurred during the pandemic (between November 2020 and August 2021). Across both communities, providers (13 in total) participated in telephone interviews, whereas interviews with stakeholders (representing 23 community organizations) occurred either in-person or by video call (Zoom) to accommodate COVID-19 requirements and providers’/stakeholders’ preferences. The interviews with providers and stakeholders occurred between June 2019 and September 2021.

The study team developed a separate semi-structured discussion guide for the focus groups and interviews for each respondent category. Questions and probes on the discussion guides were used to gain insight into participants’ general perceptions about their community and if they considered it a place to raise young children; barriers and facilitators of healthy eating and PA at home, childcare, and other community settings where children spend time; organizational and community resources, programs, and policies for supporting healthy eating and PA in children/families; and factors that promote or impede the sustainability of community programs that focus on child health. Additional questions and probes provided insight into potential intervention strategies that could be used to promote healthy eating and PA among children/families in both communities. Members of the study team who are trained in qualitative methods (MV, TE, KP) moderated these discussions. The focus groups lasted about 90 min, while interviews lasted about 45–60 min. Parents and providers completed a demographic survey, but no demographic information was collected from stakeholders.

In addition to focus groups and interviews, observational audits of the built environment were conducted to assess how well such environments supported healthy eating and PA. Food environment audits were restricted to IN because a similar assessment (unpublished data) was performed in the NC community by the Health Department in the NC community in 2016 [28]. Food outlets in the IN community were identified from internet searches using keywords such as “grocery store,” “food retailer,” and “food outlet.” Purposive sampling was used to select 2–3 food outlets per township, representing supermarkets, small markets (e.g., Dollar General), and convenience stores, for a total of 19 food outlets in the IN community. Trained data collectors (KP, research assistants) completed the Communities of Excellence in Nutrition, PA, and Obesity Food Availability and Marketing tool (CX^3^) [29, 30] at each food outlet to assess availability, quality, and marketing/promotion of healthy food items. CX^3^ entries for each food outlet were scored using an established rubric [29, 30], with higher scores representing higher-quality food environments (possible range of scores was 0–77 after excluding CX^3^ items that were not applicable to this study).

Observational audits of the built PA environment were also conducted to assess the quality of PA resources (e.g., parks, community centers) in both communities. Potential PA resources were identified from the webpages for the respective counties, Departments of Parks and Recreation, internet searches, and community drive-throughs. While the goal was to audit all no-cost, publicly accessible PA resources in both communities, the authors acknowledge that it is possible that some resources were missed and not included in the study sample. Overall, 50 PA resources were audited across both communities: 26 in IN and 24 in NC. Trained data collectors (CW, KP, research assistants) completed the Physical Activity Resource Assessment (PARA) tool [31] at each resource to assess the quality of physical features (e.g., trails) and amenities (e.g., bathrooms), and presence of incivilities that reduce the pleasure of using the resource (e.g., litter). The rubric established for scoring PARA [31] was applied to each PA resource (possible range of scores = 0–75), with higher scores indicating that a PA resource had more features and amenities, and fewer incivilities.

### Data analysis

Focus groups and interviews were digitally recorded and transcribed without identifiers. The transcripts were reviewed for accuracy and completeness, and imported into ATLAS.ti (version 3.4.5–2021-11, Berlin, Germany), a software for qualitative analysis. Members of the study team who are trained in qualitative analysis (MV, TE, CW, KP) reviewed the data and developed a codebook based on the discussion guides for the focus groups and interviews, and the study aims. The study team performed content analysis and allowed for any emergent themes to be included. Each transcript was coded by two members of the study team: a primary and secondary coder. The primary and secondary coder discussed discrepancies in the application of codes, and areas of disagreement were resolved by consensus. Parent focus groups, parent interviews, and childcare provider and stakeholder interviews examined similar topics, with several areas of overlap in participants’ responses, thus, data from each source was pooled for the final summarization of results, with quotes that illustrated each theme. The demographic survey, CX^3^, and PARA tool were analyzed using descriptive statistics (e.g., percentages, interquartile range) in R (version 4.1.1, Vienna, Austria), a software for quantitative analysis. Because this study was not meant to compare barriers/facilitators between the two communities, the study team only assessed differences in relation to participants’ demographic characteristics, which were analyzed using Fisher’s exact test (categorical variables) and t-tests (continuous variables), with statistical significance established at p ≤ 0.05 (two-sided).

## Results

### Participants’ characteristics

Overall, 41 parents, 13 providers, and stakeholders from 23 community organizations participated in this study. While the goal was to recruit male and female parents/primary caregivers of preschool-aged children, the final sample comprised solely of females who were an average of 36 years old. The majority (63%) were Black/African-American; 50% had a high school diploma or lesser qualification; 75% had resided in their communities for > 10 years; and their average body mass index (BMI) was 33 (± 9) kg/m^2^, indicating most were obese (Table 1). All providers were female, an average of 45 years old, with 54% being White, and 85% having at least some college education. Stakeholders did not provide demographic information.

**Table 1:** Demographic characteristics of parents and childcare providers from two rural communities^a^

	**Overall**	**Indiana community**	**North Carolina community**	
**Parent characteristics**	**(*n* = 41 parents)**	**(*n* = 14 parents)**	**(*n* = 27 parents)**	
	**n (%)**	**n (%)**	**n (%)**	***p*-value**^b^
Sex				
Female	41 (100)	14 (100)	27 (100)	
Marital status				
Married or living as married	19 (49)	12 (86)	7 (28)	** < 0.01**
Single, widowed, or unmarried	20 (51)	2 (14)	18 (72)	
Race				
Black or African American	26 (63)	0 (0)	26 (96)	** < 0.01**
White	15 (37)	14 (100)	1 (4)	
Yearly household income				
$50,000 or less	29 (74)	4 (29)	25 (100)	** < 0.01**
$50,001 or more	10 (26)	10 (71)	0 (0)	
Highest level of education				
High school diploma or less	20 (50)	3 (21)	17 (65)	**0.02**
Some college or Associate’s degree or higher	20 (50)	11 (79)	9 (35)	
Length of time residing in County				
10 years or less	10 (25)	6 (43)	4 (15)	0.12
More than 10 years	30 (75)	8 (57)	22 (85)	
Age ***(mean, s.d.)***	36 (12)	34 (7)	37 (15)	0.56
Body mass index (kg/m^2^) ***(mean, s.d.)***^**c**^	33 (9)	30 (9)	34 (10)	0.15
**Childcare provider characteristics**	**(*n* = 13 providers)**	**(*n* = 7 providers)**	**(*n* = 6 providers)**	
	**n (%)**	**n (%)**	**n (%)**	***p*-value**^b^
Sex				
Female	13 (100)	7 (100)	6 (100)	
Race				
Black or African American	5 (39)	0 (0)	5 (83)	** < 0.01**
White	7 (54)	7 (100)	0 (0)	
Other	1 (8)	0 (0)	1 (17)	
Yearly household income				0.77
$45,000 or less	5 (39)	2 (29)	3 (50)	
$45,001 or more	6 (46)	4 (57)	2 (33)	
Prefer not to answer	2 (15)	1 (14)	1 (17)	
Highest level of education				
High school diploma or less	2 (15)	2 (29)	0 (0)	0.46
Some college or higher	11 (85)	5 (71)	6 (100)	
Age ***(mean, s.d.)***	45 (17)	37 (12)	56 (16)	**0.04**

*Abbreviations*: *kg* represents kilograms; *m* represents meters; *s.d.* represents standard deviation

^a^Fisher’s exact test (categorical outcomes) and t-test (continuous outcomes) were calculated to examine differences in demographic characteristics between participants in the two rural communities

^b^Bolded *p*-values are significantly different at *p* ≤ 0.05

^c^Parental body mass index (kg/m^2^) was calculated based on weight and height reported on the demographic survey

### Community settings

Themes that emerged from focus groups and interviews about participants’ perceptions of their community, and barriers, facilitators, and opportunities to promote healthy eating and PA in children were generally similar across parents, providers, and stakeholders. Therefore, unless specified, such responses were captured as “participant” reports throughout this paper. Also, participants’ responses were similar across both communities, but where applicable, the authors highlighted differences. In focus groups and interviews, participants described their community using phrases such as rural, small, and poor *(both communities)*, agricultural *(IN)*, slower living, having open spaces and opportunities to connect with nature *(IN)*, majority Caucasian *(IN)* or racially-diverse *(NC)*, and religious *(both)*. Both communities were described as friendly, safe places to raise children, and community-minded (i.e., a place where residents knew and looked out for one another), but participants also highlighted concerns about crime and substance use in some neighborhoods. A stakeholder shared: *“I do like the small-town feel. You know, the fact that I can let my kids run around and play in town… You know, the community looks out for everybody’s kids. So, I do appreciate that. It’s definitely not, you know, all bad by any means. We have our barriers, but doesn’t everyone?”.*

In audits of the built environment in the IN community, 19 food outlets were assessed, including 3 supermarkets, 10 convenience stores, and 6 small markets (Table 2). Seventy-four percent (74%) accepted SNAP (Supplemental Nutrition Assistance Program), while 21% accepted WIC benefits. More than half of the food outlets were rated as having limited or no variety of fresh fruits (58%) or vegetables (63%). Over half were also rated as having poor quality or no fresh fruits (68%) or vegetables (58%). The median of the total score for the food environment (based on CX^3^) was 18 out of 77, indicating there are opportunities to improve access to healthy foods in the built food environment.

**Table 2:** Results from the assessment of food outlets in the rural Indiana community using CX^3^ tool^a^

	**Indiana Community**
	**(*n* = 19 food outlets)**
	**n (%)**
**Type of food outlet**	
Supermarket	3 (16)
Convenience store	10 (53)
Small market	6 (32)
**Variety of fresh fruits in store**	
None or limited variety	11 (58)
Moderate or wide variety	8 (42)
**Variety of fresh vegetables in store**	
None or limited variety	12 (63)
Moderate or wide variety	7 (37)
**Quality of fresh fruit in store**	
No fresh fruit or poor quality	13 (68)
Mostly good or all good quality	6 (32)
**Quality of fresh vegetables in store**	
No fresh vegetables or poor quality	11 (58)
Mostly good or all good quality	8 (42)
**Accepts Supplemental Nutrition Assistance Program (SNAP) benefits**	
Yes	14 (74)
No	5 (26)
**Accepts Women, Infants and Children’s (WIC) program benefits**	
Yes	4 (21)
No	15 (79)
**Availability of other healthy food items (e.g., whole wheat bread, skim milk, brown rice, frozen vegetables)**^**b**^ (*median, IQR)*	7 (6)
** Total food environment score on the CX**^**3**^** tool**^**c**^*(median, IQR)*	18 (43)

*Abbreviations*: *IQR* represents interquartile range

^a^Food environment audits were restricted to IN because a similar assessment was performed in the NC community by the Health Department in the NC community

^b^Possible range of scores = 0–10; scores greater than 8 meet health recommendations

^c^Total scores on the CX^3^ tool could range from 0–77, with higher scores representing higher-quality food environments

In audits of the built PA environment, 50 community resources were assessed, including nine parks/trails, five sports facilities, 18 school playgrounds, and 18 locations that were a combination of PA resources (e.g., sports facility with a playground) (Table 3). Median scores for PA features, amenities, and incivilities (based on PARA) were 12 (out of 39), 15 (out of 36), and 2 (out of 36) respectively. The median of the total score for the PA environment score was 21.5 out of 75, indicating there are opportunities to improve access and the quality of PA resources in the built PA environment.

**Table 3:** Results from assessments of physical activity resources in two rural communities using the PARA tool

	**Overall**	**Indiana**	**North Carolina**
	**(*n* = 50 resources)**	**(*n* = 26 resources)**	**(*n* = 24 resources)**
	**n (%)**	**n (%)**	**n (%)**
Type of physical activity resource			
Park or trail	9 (18)	5 (19)	4 (17)
Sports facility	5 (10)	5 (19)	0 (0)
School playground	18 (36)	8 (31)	10 (42)
Combination of the above resources	18 (36)	8 (31)	10 (42)
	**median (IQR)**	**median (IQR)**	**median (IQR)**
Features score of physical activity resources audited^a^*(range: 0–39)*	12 (11.5)	16 (6)	10 (6)
Amenities score of physical activity resources audited^a^*(range: 0–36)*	15 (10.5)	17.5 (8.8)	9.5 (9)
Incivilities score of physical activity resources audited^a^*(range: 0–36)*	2 (3.5)	2 (3)	2 (3)
Total PARA Score^b^*(range: 0–75)*	21.5 (15.5)	30 (19.5)	17.5 (7.3)

*Abbreviations*: *PARA* represents physical activity resource assessment tool, *IQR* represents interquartile range

^a^Features, amenities, and incivilities received scores ranging from 0–3 (0 = not present; 1 = poor; 2 = mediocre; and 3 = good)

^b^Total PARA score = Features + Amenities – Incivilities

### Barriers to healthy eating and physical activity

Barriers to healthy eating and PA that emerged from discussions with parents, providers, and stakeholders centered around four main themes: parental practices related to nutrition; limited financial resources within families; limited availability and access to health promotion programs; and limited access to community resources that can support healthy eating and PA in children (Table 4). At the family level, unhealthy nutrition practices were described as occurring because of parents’ limited cooking skills and lack of nutrition knowledge. A stakeholder explained: *“You know, so a lot of people don’t even know how to make meals out of the actual food that’s not pre-packaged with an oven. You know, instructions on the side.”* Limited financial resources posed a challenge, especially for single parents, augmented by the perception that eating healthy was “expensive” *(stakeholder)*. Related to this were competing priorities, including navigating lack of childcare/support networks, paying bills, and parental mental health concerns; these prompted many parents to prioritize providing basic needs, use fast food frequently, and rely on electronics to entertain children. A provider shared: *“They’re all working you know, low pay, long hours. They are kind of burnt out already.”*

**Table 4:** Barriers to healthy eating and physical activity in preschool-aged children in two rural communities

Main Theme	Sub-Theme	Illustrative Quotes
**Family level**		
Unhealthy nutrition practices	Parental lack of cooking skills	“People don't know how to cook anymore. They just don't know how to cook, and they don't believe even if they do know how to cook, they don't believe they have the time to cook.” **(Stakeholder)**
	Parental lack of nutrition knowledge	“There's definitely a lot of ignorance about nutrition in general, where like ‘Juicy Juice’ says the word juice in it, therefore it’s fruit, this counts. And it’s not understanding how nutrition actually works. And therefore, thinking that they are getting fruits and vegetables when they aren't.” **(Parent)**
Limited financial resources within families	Perception that healthy foods are expensive	“Cost is a huge barrier to healthy food choices. And then the lack of where you would even go to get it outside of traveling farther away… People are compartmentalized, but there's one overarching thing and that is: how can I survive. Period. And so, these decisions about better foods as opposed to less expensive foods… what's going to win is always going to be less expensive foods.” **(Stakeholder)**
	Other priorities competing with eating healthy and being active	"A lot of these parents are doing everything they can just to get food on the table. If they're having trouble with food, having their kid go out and exercise is not a priority.” **(Parent)** “Looking at the expenses that you have, healthy food is going to go below rent. So as long as you have any kind of food, that is going to be your priority.” **(Stakeholder)**
**Organization level:**		
Limited availability/access to health programs	Limited access to childcare programs	“Childcare is definitely a huge barrier. If you don't have an immediate family member who can stay with your kids, then, your options are pretty limited.” **(Parent)**
	Perception that practices at childcare are not reinforced at home	“We follow food programs, so we follow more strict nutritious guidelines. But you know, if they leave and go straight through Wendy's drive-thru, all my work is for naught. And I know there's not anything you can do about that. You can't force a parent to treat them differently.” **(Childcare provider)**
	Poor parental engagement in health programs	“It’s hard. Parent involvement, getting parents involved, is our biggest downfall.” **(Stakeholder)**
**Community level:**		
Limited access to community resources	Limited access to healthy foods (and beverages) and healthy food outlets	“It's the access. So, we tend to go to Dollar General like in a pinch. Like we need peanut butter, and we just need to get it right away for a recipe or something. But it’s nice to go there when we don’t feel like cooking, or we just want to get something for the kids. I would like to have a little farmers’ market or something there that we could, where we could get fresh fruits and vegetables. And so quick access, I would say is tough.” **(Parent)** “I mean we have clean water, there are parts of our county that don’t probably have running water. And I say that’s not a majority, but then again there definitely are pockets of the county that are in such poverty conditions that. That is part of being so spread out. So, there’s pockets here where there is just not access.” **(Stakeholder)**
	Limited access to physical activity resources	“We don't have a park. That's one thing that has really bothered me a lot. The only park in eastern [county] is the playground at the school… we can go there as long as school’s out. Of course, all the kids who are schooling remotely can't go there during the day… It's kind of hard. Especially we've got … essentially just the eastern third of the county: the only park is at the school.” **(Parent)** “There are things there that the children can use… There's a walking track and there's swimming but you have to pay to swim. Now, you know, we…. the county does try, but not for the preschoolers, of course, to have sports, basketball, football, soccer, things like that, and they may even have some for five-year-olds, but that would probably be the youngest. But we don't really have child-friendly or toddler-friendly places here.” **(Stakeholder)**
	Safety concerns within neighborhoods	“There is a basketball court in the park, but you never see kids there. You never see anybody at the park, and I just don’t know that it’s—it’s perhaps not safe or the kids are doing other things. But the town is not as safe as it used to be.” **(Stakeholder)**
	Lack of public transportation	“We don't have any public transportation… So, if folks don't have their own transportation, they would have to rely on a support system. You know grandma or neighbor whatever.” **(Stakeholder)**

Organizational level barriers centered around limited availability and access to health promotion programs. Parents discussed having few childcare programs in their community *(IN)*. While providers discussed having to comply with strict nutrition and PA guidelines, they perceived that related messages/practices were not reinforced at home. Childcare providers reported lack of funding as preventing them from investing in more nutrition and PA education/resources. While community organizations reported offering health promotion initiatives, poor family engagement and poor outreach were described as challenges. A stakeholder shared, “*Yeah, so they (parents) miss out on a lot of opportunities, um, because they won’t come out. So, we are trying to find ways to get the parents to come out.”*

At the community level, limited access to community resources that could support healthy eating and PA in children were described as challenges. In particular, participants voiced concerns that few grocery stores carrying fresh, healthy, and inexpensive foods were available, and of the options that did exist, their shelf-life was relatively short. Parts of both communities lacked access to clean water. While some PA resources (e.g., parks) were available in the communities, remote areas were described as lacking access. Because both communities lacked public transportation, parents without vehicles relied on family/friends for transportation, thus, restricting their ability to engage children in activities beyond the home, such as organized sports. Voicing this concern, a parent shared, *“From what I have heard, some parents may not have access to vehicles all the time to be able to even take their children to participate in activities.”* Concern for safety, because of violence and substance use in some neighborhoods, prevented some parents from allowing outdoor play. Group-based physical activities for preschool-aged children were limited and described as costly.

### Facilitators of healthy eating and physical activity

Facilitators of healthy eating and PA focused on three main themes related to: parental efforts to promote healthy eating in children; ongoing efforts by providers and stakeholder organizations to promote nutrition, PA, and health; and existing assets/resources for promoting nutrition, PA, and health in the community (Table 5). At the family level, participants described efforts by some parents to role-model and prioritize the provision of healthy foods at home. A parent shared: *“We tend to have a large bowl full of fruits (sic) and some in the refrigerator at all times. But when we decided to do that, we had to shift our minds and our budget because they are more expensive. But our kids eat them much more now.”* Echoing similar practices, another parent said, *“I give him (child) healthy things, I don’t give him sweets and sugar, and stuff, even though it be what he wants.*”

**Table 5:** Facilitators of healthy eating and physical activity in preschool-aged children in two rural communities

Main Theme	Sub-Theme	Illustrative Quotes
**Family level:**		
Efforts by parents to promote healthy eating in children	Role-modeling by some parents	“I have learned that what you do for children will follow. Like they will follow whatever you do because they think is right. Your mom, like you know, they think that what (you do) is golden.” **(Parent)**
	Efforts to provide healthy foods by some parents	“So, we value trying new foods and serving healthy foods as much as possible. Sugar-free, low-carb, high in protein and healthy fats. And I would say that we eat from a variety of cultures and prepare foods from a variety of cultures.” **(Childcare provider)**
**Organization level:**		
Ongoing health promotion efforts in the community	Health promotion efforts at childcare programs	“I do try to encourage more whole foods because it's so much better for you. And the kids try it, they'll try at the daycare. They're encouraged when I encourage them to try something new or they see me eating it or then well she eats it, [*so I’ll eat it.*]” **(Childcare provider)** “Plus, we have the cafeteria that provides a meal. Right now, they're offering free lunch, free breakfast, and also, something that they call dinner bags for all of our kids right now. That is something great… They try to send a fruit and a vegetable daily, along with a protein and a dairy item.” **(Childcare provider)**
	Health promotion efforts through other community organizations	“We’ve also had a recent partnership … where we provided nutrition support classes. All of these programs, again, it's not just about throwing food at people but it's about making sure that people have things like sharp knives so they can process, they can actually cut fresh produce. Making sure they have cutting boards; making sure they know how to store onions versus potatoes so that they last longer.” **(Stakeholder)**
**Community level:**		
Existing community assets and resources	Existing resources in the community that can support healthy eating and physical activity	“The park is owned by the city; it is utilized a lot... . So, there are festivals held there. Just about any kind of community event is held there. This is pre-COVID of course. There is also the Farmers’ Market held every Saturday from May until September… it is definitely our biggest area for fitness/physical activity.” **(Stakeholder)** “[County] has an excellent Farmers’ Market, so I try to promote those as much as I can. Because there are good prices. You can go and get fresh fruits and vegetables at not a bad price at the Farmers’ Market. Promoting those things that, I think, in everybody’s mind, if you eat healthy it’s going to be super expensive because you have to go organic in order to eat healthy, but that’s not necessarily true.” **(Stakeholder)** “The hospital definitely promotes healthy living and physical activity. Like I said before, we are connected with the park, and we try to strengthen the park as much as we can. We offer grant support; we help facilitate the grant that they just received this year.” **(Stakeholder)**

Facilitators at the organizational level centered on health promotion initiatives offered through childcare programs and community organizations. Childcare programs were described as providing nutritious meals to children at no-cost to families. Providers described offering a variety of foods, creating opportunities for children to try new foods, and serving as role-models. Providers also described growing fruit and vegetable gardens with children, and providing nutrition lessons, diverse opportunities for children to be physically active, and health education activities/resources to families. Participants described childcare programs and community organizations as helping to foster relationship building and an avenue for resource-sharing among families. Additional community-specific assets were described; for example, in the IN community, residents could access nutritious foods through food pantries, Blessing Boxes, and doubling of WIC/SNAP benefits at farmers’ markets (Market Bucks). In the NC community, participants cited a mobile food market, ongoing efforts to connect childcare programs with locally grown food, and some PA programs offered at low-cost through the community center.

Facilitators/assets at the community level focused on available nutrition and PA resources (e.g., farmers’ markets, parks). Participants appreciated ongoing investment by the county and private entities to improve these resources and create new PA infrastructure and health promotion programs for their community.

### Opportunities to promote healthy eating and physical activity

Participants described factors to consider when designing/implementing a community-based intervention to promote healthy eating and PA in preschool-aged children. In doing so, they discussed the need to garner the community’s trust; target the entire family instead of focusing solely on children; and promote interventions that focus on overall wellness. A stakeholder said: *“People gotta’ be with, they gotta’ be willing to trust you… You got to make yourself available. You can’t say, “well okay, I’m coming next week,” and then, you don’t show up.”* Additionally, developing partnerships with community organizations, and leveraging of existing community resources were recommended as potential intervention targets.

According to participants, other intervention considerations could include providing an educational component to promote familial awareness about healthy habits, using local experts to provide education/training, and conducting direct outreach to families to promote awareness about potential interventions. Improvement of the public transportation system so that residents are able to access resources without a personal vehicle, and continued investment in community infrastructure (e.g., grocery stores, parks) to increase access to healthy foods and PA resources were recommended. Creation of family-friendly, wellness-focused, affordable community events to community residents was recommended. Voicing this, a parent said: *“I think we need more things to do in the community, not just waiting for the fair once a year… I believe parents and children should have more interaction together. Not children do this, and then the parents go do that.”* Stakeholders highlighted the importance of creating policies to support healthy dietary and PA habits in children/families.

## Discussion

This paper describes barriers, facilitators, and opportunities to promote healthy weight behaviors in preschool-aged children in two rural communities, a topic on which little research presently exists [12]. Similar to previous studies [3, 9, 10, 32], the authors found that participants were generally ambivalent about their community’s attributes, often appreciating the small, rural, slow-paced and close-knit community feel, with access to open spaces and nature, but bemoaning the high poverty levels and built environment barriers. Notably, participants’ reports about the built environment barriers related to nutrition and PA (Table 4) were supported by findings from the audits that showed the need to improve access to healthy foods (Table 2) and high-quality PA resources (Table 3), and consistent with findings of other studies of older children and adults in rural communities [33–35]. While food environment audits in this study were restricted to IN, the results were similar to a recent assessment of 13 food outlets in the NC community by the Public Health Department (unpublished) in which the median CX^3^ score was 16 out of 77 (versus 18 out of 77 for IN). Other community-level challenges reported by participants, including lack of transportation, and concerns about physical safety while outdoors, have been reported in other studies of rural communities [10, 33, 36, 37].

At the family level, barriers included parental lack of nutrition knowledge, limited cooking skills, and constrained financial resources coupled with competing priorities, consistent with other studies of preschool-aged children [12]. When health promotion programs were offered, providers and stakeholders said it was difficult to engage parents of young children in such activities. Paradoxically, many parents reported a lack of community resources for supporting healthy lifestyle behaviors. Despite the challenges reported, participants cited many facilitators/assets in their community to support healthy choices. For example, at childcare programs and community organizations, health promotion programs (e.g., nutrition education lessons) and resources (e.g., Blessing Food Boxes) were offered to families. Community generosity was frequently mentioned as an asset, and community events (e.g., county fairs) offered opportunities for families to recreate together; again, reinforcing the importance of kinship in rural communities [2, 3]. Several participants lauded adult modeling as an effective way to encourage children to make healthy dietary choices in and outside of the home, which research shows is a critical component of childhood obesity prevention interventions [7, 12].

Participants in this study were solution-oriented, suggesting factors for the study team to consider when developing an intervention to promote healthy eating and PA in children. They recommended that the study team establish joint partnerships with local community organizations, leverage existing community resources, and take the time to establish trust with community members. Improvements to existing infrastructure (e.g., parks), including provision of transportation options for persons without personal vehicles, and offering of more community activities targeted toward the entire family were recommended; these potential solutions are promising, but their impact on children’s nutrition and PA behaviors have been understudied [7, 12, 37]. Other researchers recommend that rural communities consider investing in improving existing resources by developing joint-use agreements with stakeholder organizations (e.g., schools, local farmers) to better share resources/facilities, create policies that enable easier access to nutrition and PA resources, and address challenges associated with lack of public transportation [37].

In the current study, most data from NC were collected before the COVID-19 pandemic, whereas data collection in the IN community occurred during COVID-19, which accounted for the use of different modes of data collection with parents (focus groups conducted in-person in NC versus interviews by telephone or video call in IN) and could have impacted participants’ perceptions about barriers and assets/facilitators. The study findings may not be generalizable to all rural U.S. communities, but the inclusion of diverse perspectives from parents, providers, and stakeholders is a strength. While the goal was to recruit both female and male caregivers/parents of children aged 2–5 years, only female caregivers/parents participated in the study. The use of convenience sampling to recruit parents, providers, and stakeholders is considered a limitation. As noted by other researchers [9], it is possible that participants in the current study were persons who were more conscious about health. Nevertheless, the use of multiple methods, including focus groups, interviews, and audits to collect data is considered a strength. Additionally, the tools used for observational audits of the built food and PA environment (CX^3^, PARA) have demonstrated evidence of reliability in other studies [29, 31, 38].

## Conclusions

The high child obesity levels in rural communities are concerning, and unhealthful dietary intake and physical inactivity are modifiable behaviors that increase the risk for childhood obesity [5, 6]. This study highlights barriers that prevent rural children from engaging in healthy eating and PA behaviors, including unhealthy nutrition practices related to parents’ limited cooking skills and nutrition knowledge, and poor access to health promotion programs and community resources that promote healthy weight. Study participants also highlighted several facilitators/assets, including ongoing health promotion programs and existing community resources (e.g., parks) that can support healthy child weight. Findings will guide the study team in developing a multi-level community-based intervention to promote healthy weight behaviors in rural preschool-aged children. Findings highlight the need for policy and interventions (e.g., nutrition education for families, improvements to the built environment to increase access to healthy foods and PA resources) to promote healthy eating and PA behaviors in children/families in the target rural communities.

## Data Availability

The datasets generated and/or analyzed during the current study are not publicly available because the study team would like to know and keep track of how the data will be used by others; however, the datasets can be made available by contacting the corresponding author on reasonable request.

## Abbreviations

BMI: Body mass index
CX^3^: *Communities of Excellence in Nutrition, PA, and Obesity* Food Availability and Marketing Survey
IN: Indiana
NC: North Carolina
PA: Physical activity
PARA: Physical Activity Resource Assessment
SNAP: Supplemental Nutrition Assistance Program
WIC: Special Supplemental Nutrition Program for Women, Infants, and Children
